# Simulation and Optimization of Transmitting Transducers for Well Logging

**DOI:** 10.3390/s24216795

**Published:** 2024-10-23

**Authors:** Xu Gao, Jing Zhou, Xiao Du

**Affiliations:** 1 College of Petroleum Engineering, Xi’an Shiyou University, Xi’an 710065, China; 20111010002@stumail.xsyu.edu.cn (X.G.); 22111010007@stumail.xsyu.edu.cn (X.D.); 2 National Engineering Laboratory for Oil and Gas Drilling Technology, Xi’an Shiyou University, Xi’an 710065, China

**Keywords:** microporous electrode, transmitting transducer, liquid–electric

## Abstract

Piezoelectric transducers are commonly used in acoustic well logging. However, the low frequency and narrow range of the acoustic waves limit the achievable detection accuracy. In addition, the low amplitude of the waves causes useful information to be easily masked by noise during detection, which affects the accuracy of geological identification and makes it difficult to detect formations tens of meters away. This paper proposes a microporous liquid–electric transmission transducer, in which the microporous electrode structure generates a powerful shock wave through a high-energy instantaneous discharge. First, a model of the liquid–electric microporous transmitting transducer was constructed by combining simulations with numerical calculations, and its electro-acoustic characteristics were analyzed. Then, based on the survey requirements, two innovative optimization schemes for the microporous electrode structure were proposed, namely a triangular pyramid microporous electrode structure and a rectangular microporous electrode structure, and their performances were compared. The results show that the newly optimized triangular pyramid microporous electrode liquid–electric transducer generates acoustic waves with higher amplitude and a wider frequency range than conventional piezoelectric transducers and other microporous structures. It maintains high energy while achieving high frequencies, enabling detection at distances of up to hundreds of meters and the precise characterization of small geological bodies. This has significant implications for applications in marine exploration, land exploration, clean energy, and new energy fields.

## 1. Introduction

With the continuous consumption and extraction of oil and gas resources, the exploration and development of some oil and gas fields have entered the mid-to-late stage, and the difficulty of extraction has increased. Correspondingly, logging technologies have evolved, and there is an urgent need to break through traditional oil exploration techniques in order to extend the life cycle of the petroleum industry [1]. The key challenge for the next generation of acoustic logging tools is to detect oil and gas resources at greater distances and with higher detection resolution [2,3].

In recent years, acoustic logging technology has been widely used in oil and gas exploration. Acoustic logging uses acoustic waves generated by transmitting transducers as incident waves, while receiving transducers detect the signals reflected and refracted by the geological formations downhole [4]. By processing and analyzing these signals, geological parameters can be obtained [5,6], allowing for the inversion and imaging of geological structures [7], the assessment of cement bond quality [8], well trajectory optimization, and three-dimensional (3D) reservoir evaluation, among other applications [9,10].

The factors that affect the detection resolution and range of acoustic logging technology mainly include the performance of the acoustic transmitting transducer, the sensitivity of the receiving transducer, the propagation loss of the waves in the formation, and other factors. Among these, improving the performance of the acoustic transmitter is the most direct and effective approach [11]. The sound pressure intensity and frequency of the transmitting transducer directly affect the detection range and resolution of the logging process. The greater the amplitude of the sound wave and the lower the frequency, the greater the detection range, but the lower the resolution [12].

In recent years, the development of acoustic logging technology has been accompanied by advances in acoustic transmitting transducers. There are four main types of transmitting transducers, as follows:

(1) Monopole Transmitting Transducers: The operating frequency of monopole transducers is typically around 10 kHz. Due to rapid attenuation during propagation through the formation, these transducers can only detect structures within a few to several meters of the borehole. In soft formations, they cannot provide shear wave information. Monopole transmission can only determine the distance of the reflector from the source, not the azimuthal direction of the reflector [13,14].
(2) Dipole Transmitting Transducers: Multicomponent dipole transmitting and receiving transducers can determine the location of reflectors [15]. The operating frequency of dipole transducers is typically between 2 kHz and 5 kHz [16], allowing lateral detection depths of tens of meters. The sound field forms a figure-of-eight pattern in the plane, providing some directionality. However, there is an azimuthal uncertainty within a range of 180°, which limits the use of dipole transducers to some extent [17].
(3) Phased Array Transmitting Transducers: Phased array transducers are formed by arranging multiple transducers in a specific pattern. Common configurations include phased linear arrays and phased circular arc arrays [18]. The operating frequency of phased array transducers is approximately 14 kHz [19]. The phased array allows directional sound wave radiation, which significantly improves detection capabilities and azimuthal resolution [20]. However, the power and frequency range of phased array transducers is limited, and their detection range is limited to tens of meters [21]. In addition, related instruments are still at the experimental stage and the further development of mature processing and interpretation methods is required.
(4) Multipole Transmitting Transducers: Multipole transducers combine the characteristics of monopole, dipole, and quadrupole transducers [22]. The operating frequency range is between 15 kHz and 25 kHz, and they can generate a three-dimensional sound field within tens of meters of the borehole [23]. However, the sound wave intensity of multipole transducers is relatively low, resulting in a low signal-to-noise ratio. This causes useful acoustic signals carrying information about geological structure to be disturbed or masked by noise, which interferes with normal measurements and drilling operations [24,25].

The monopole, dipole, multipole, and phased array transducer types commonly used in logging are all traditional piezoelectric transducers [26,27]. The natural frequency of a piezoelectric transducer is related to its volume and cannot be changed once the transducer is manufactured. The limited space in the borehole limits the size of the transducer, resulting in a narrow frequency range for conventional transducers, which are unable to operate effectively at both high and low frequencies [28]. In addition, the frequency range is typically between 0 and 25 kHz [29], which limits the detection resolution. The sound pressure level is relatively low, making it susceptible to attenuation within the formation, and easily drowned out by unwanted noise. The detection range is only a few tens of meters around the borehole, making it difficult to accurately identify reflectors at greater distances.

In recent decades, with the increase in research on the mechanism of high-voltage pulsed water discharge and related technologies, a large number of scholars have focused on the innovation of electrode structures in research on hydroelectric converters. The electrode structure is becoming more diversified. In addition to the traditional combination of rod electrode structure [30], needle electrode structure [31], and ring electrode structure [32], in 2004, Wang Jing [33] of Wuhan University adopted a novel microporous electrode to study the solution extraction mechanism. Zhang et al. [34,35] systematically investigated the discharge characteristics of microporous electrode structure, and pointed out that the microporous electrode possessed a high amplitude of acoustic wave, and it was applied in marine exploration.

This paper proposes the use of liquid–electric transducers in the field of acoustic logging. Liquid–electric transducers are characterized by their small size and high power output. The underlying theory is based on the liquid–electric effect, where the energy stored in a capacitor is applied across the electrode gap in an extremely short time, generating a powerful acoustic wave in the fluid medium. The essence of the liquid–electric effect is energy conversion, where electrical energy is converted into thermal energy, acoustic energy, light energy, and other forms of energy. The sound pressure intensity generated by these transducers is significantly higher than that of conventional piezoelectric transducers, allowing longer propagation and detection distances. The wide frequency range of liquid–electric transducers improves detection accuracy and, when fitted with a focusing shield, they can achieve directional and focused emissions. In addition, their small size makes them easy to install on drill collars [36].

First, a simulation model of the liquid–electric micro-pore transducer was constructed using COMSOL Multiphysics 5.6 software. A comprehensive analysis of its electrical and acoustic characteristics was performed in conjunction with numerical calculations. Then, based on the actual survey requirements, two innovative optimization schemes for the microporous electrode structure of the liquid–electric transducer were proposed to further improve its electro-acoustic performance, making it more suitable for well logging and providing a reference for the design of liquid–electric transmitting transducers. This approach facilitates the detailed characterization of shale fractures, small oil and gas traps, and microfractures, enabling accurate 3D reservoir description and comprehensive evaluation over a wide area.

## 2. Methods

### 2.1. Principle

**Figure 1:**
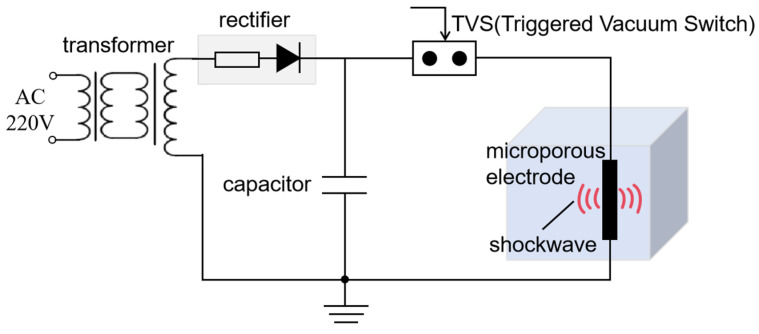
Working principle of the liquid–electrode micropore transmitting transducer.

Figure 1 illustrates the working principle of the liquid–electrode micropore transmitting transducer. The micropore electrodes are immersed in water. When the trigger control switch is activated, the 220 V alternating current is transformed and rectified to produce a high supply voltage. The capacitor continuously stores energy. As soon as the trigger control switch is deactivated, the considerable energy stored in the capacitor is immediately released via the two ends of the micropore electrodes. This rapid release of energy causes the liquid between the micropore electrodes to break up, creating a shockwave discharge. This discharge phenomenon is known as the “liquid–electric effect” [37].

The discharge process of the liquid–electrode micropore transmitting transducer in water can be divided into three sequential stages: the pre-breakdown stage, the breakdown stage, and the main discharge stage. The pre-breakdown stage is the initial phase of the discharge in water, where the discharge electrodes generate a flow under the influence of the electric field. In the breakdown stage, this flow extends and develops until it bridges the gap between the electrodes, causing breakdown and forming a plasma channel. During the main discharge stage, the high-temperature, high-pressure plasma channel expands, generating a shockwave due to the incompressibility of the surrounding liquid medium.

### 2.2. Theory

For the purpose of facilitating research and computational analysis, the plasma channel generated by the discharge is simplified to a cylindrical shape, as proposed by Kratel, to model the plasma channel of the micropore electrode [38]. The cylindrical channel of the micropore electrode has a radius of a, with the channel pressure and temperature denoted as P and T, respectively, and the current passing through the electrode gap as I. Assuming the channel behaves as an ideal gas, the shockwave pressure generated by the breakdown can be calculated using the ideal gas law [39], as follows:

(1) $$P=nkT-\frac{\mu_{0}I^{2}}{8\pi^{2}a^{2}}-\frac{e^{2}}{32\pi^{2}\varepsilon_{0}}{\left(\frac{4\pi n}{3}\right)}^{4/3}$$

where n, k, and $\varepsilon_{0}$ are constants, $\mu_{0}$ is the vacuum permeability, and e is the unit power.

The strength of the shockwave is described using the sound pressure level (SPL), which is calculated as follows:

(2) $$SPL=20\mathrm{lg}\frac{P}{P_{ref}}$$

where $P_{ref}$ is the reference sound pressure.

The shockwave at a horizontal distance of x meters from the center of the discharge electrode is given by the following equation:

(3) $$P_{r}=\frac{P\cdot a}{x}$$

where a is the channel radius and x is the horizontal distance of the impulse wave probe from the center of the discharge electrode.

The total energy discharged in the liquid by the transmitting transducer, which is the energy stored in the capacitor, is given by the following equation:

(4) $$E_{c}=\frac{1}{2}CU_{c}^{2}$$

where C is the energy storage capacitor and $U_{c}$ is the capacitor charging voltage.

Thus, the energy of the shock wave can be expressed as follows:

(5) $$E_{w}=\frac{4\pi x^{2}}{\rho c_{s}}\int {P_{r}}^{2}dt$$

where ρ is the density of the water medium, $P_{r}$ is the shock wave pressure, and $c_{s}$ is the speed of sound in the water.

The conversion efficiency of the total energy discharged in the liquid by the transmitting transducer into shockwave energy, known as the electroacoustic efficiency, is given by the following equation:

(6) $$\eta_{1}=\frac{E_{w}}{E_{c}}\times 100\%$$

where $E_{c}$ is the initial energy storage of the capacitor and $E_{w}$ is the shock wave energy.

### 2.3. Factors

**Figure 2:**
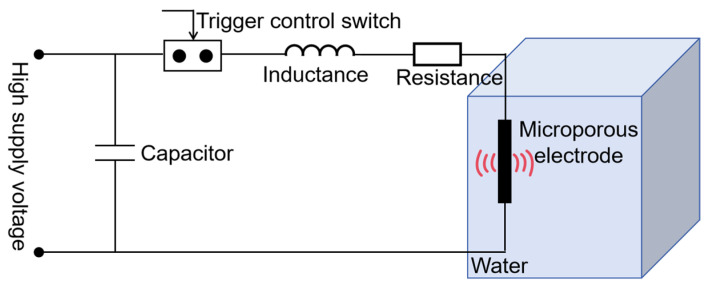
Equivalent circuit diagram for the discharge of a microporous transmitting transducer.

As shown in Figure 2, the overall circuit of the microporous transmitting transducer was simplified to an equivalent circuit during model construction, with the microporous electrodes fixed in the center of the water tank. The water tank was filled with tap water and had a side length of 40 cm. The circuit and water parameters are given in Table 1. The initial temperature of the tap water was 293.15 K. The cathode and anode of the electrodes were connected to the output terminals of the circuit, with a charging voltage of 20 kV across the capacitor.

## 3. Modeling

### 3.1. Micropore Electrode

**Figure 3:**
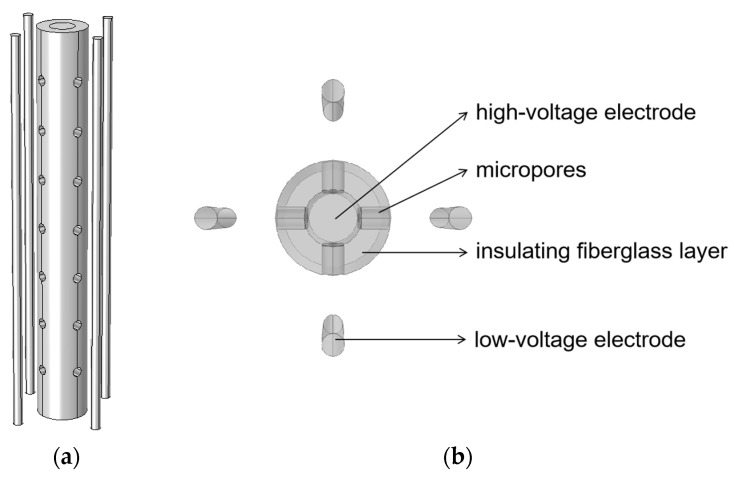
Microporous electrode model diagram: (**a**) overall view; (**b**) top view.

A micropore electrode model was constructed, as shown in Figure 3. The central cylinder represented the stainless steel high-voltage electrode. The high-voltage electrode was wrapped in an insulating layer of glass fiber [40], with four rows of micropores arranged circumferentially along the insulation. Each row contained seven cylindrical micropores, evenly spaced and coaxially aligned. Directly opposite each row of micropores were four stainless steel rods distributed around the outside of the high-voltage electrode, acting as low-voltage electrodes. The corresponding dimensional parameters of the micropore electrode model are listed in Table 2. The micropore electrode was placed in a water tank connected to an equivalent external circuit. The circuit and water parameters are given in Table 1.

### 3.2. Results

**Figure 4:**
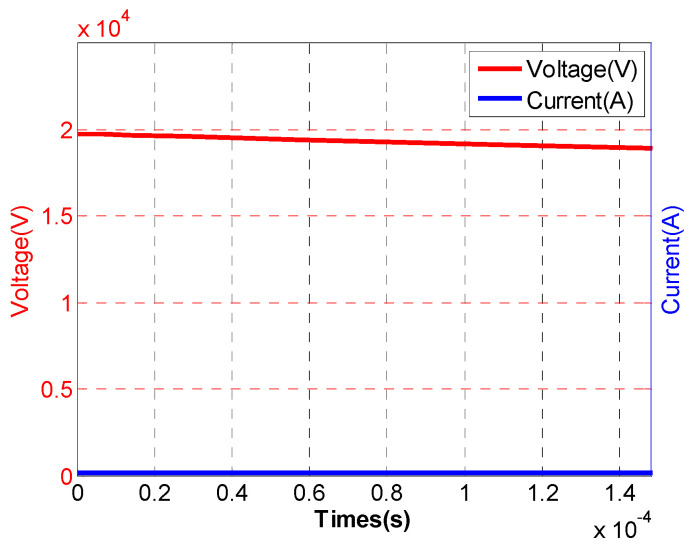
Voltage and current at different moments during the pre-breakdown process of the microporous electrode.

**Figure 5:**
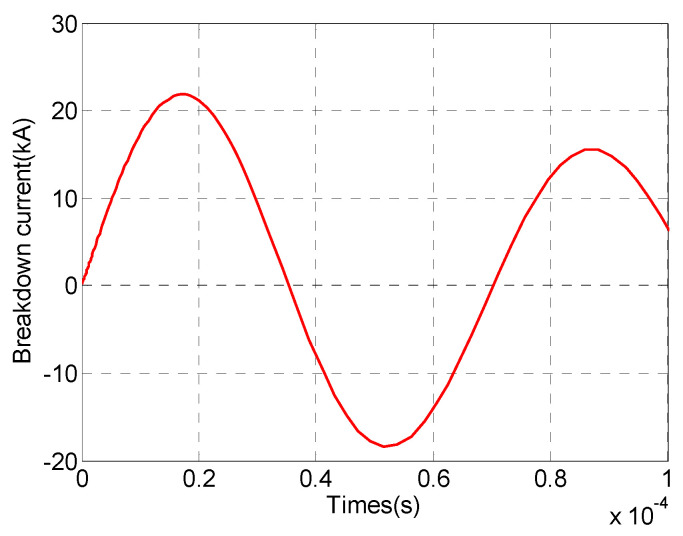
Discharge current during the discharge process of the microporous electrode.

**Figure 6:**
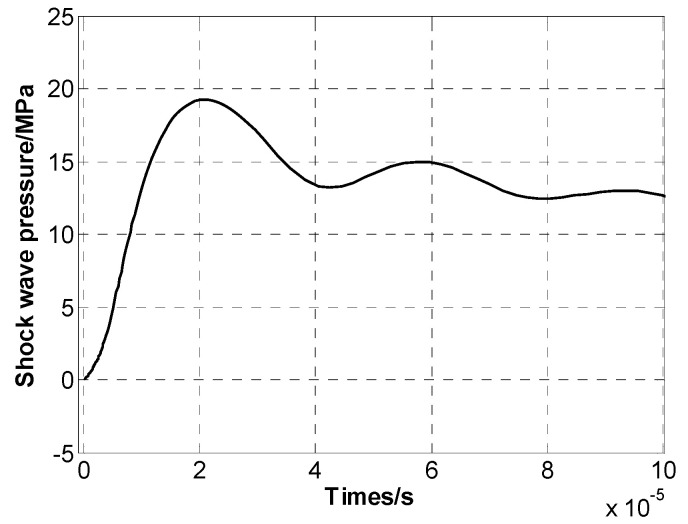
Pressure–time diagram of the shock wave generated by the microporous electrode.

A micropore electrode model was established using finite element simulation software, with electrodes made of stainless steel. In the simulation, the equivalent circuit described in Section 2.3 replaced the external circuit for the discharge of the liquid–electric transducer, where the output part of the circuit was connected to the high-pressure and low-pressure electrodes of the electrode structure to provide voltage. The micropore electrode was fixed in a cubic water tank with a side length of 40 cm, filled with tap water. The circuit parameters and water parameters are listed in Table 1. A charging voltage of 20 kV was applied to the external circuit of the micropore electrode, leading to the breakdown of the micropore electrode, while real-time monitoring of the stored voltage, current, and other electroacoustic parameters was conducted. The simulation study analyzed the electroacoustic characteristics of the micropore electrode, and the voltage and current profiles at different times during the pre-breakdown process, the discharge current profile, and the shock wave pressure–time profile were plotted, as shown in Figure 4, Figure 5, and Figure 6, respectively.

As shown in Figure 4, a charging voltage of 20 kV was initially applied to the micropore electrode, after which the voltage gradually decreased until breakdown occurred at 18.66 kV. The entire pre-breakdown process of the micropore electrode lasted 148.01 μs. From Figure 5, it can be observed that the peak discharge current reached 21.86 kA, followed by an oscillating decay. Figure 6 shows that the maximum shockwave pressure was 19.26 MPa.

## 4. Improvement

In order to investigate electrode structures capable of generating stronger shock waves, shock wave pressure and bandwidth are critical design parameters in the development of liquid–electric transducers. The higher the shock wave pressure and the wider the bandwidth, the greater the detectable range of geological formations surrounding the borehole, leading to improved performance in practical engineering applications. The electrode’s structure is a key factor influencing the characteristics of the liquid–electric transducer. Improving the microporous electrode structure to generate higher pressure and wider bandwidth shock waves is therefore essential.

As the curvature of the electrode head increases, a more distorted electric field is generated between the electrodes, resulting in a higher degree of electric field non-uniformity [41]. This non-uniform field can significantly shorten the breakdown time and reduce power consumption, thereby achieving higher shock wave pressure. In addition, the corrosion resistance of the microporous electrode and the high shock wave pressure advantages of the needle plate electrode can be combined. Therefore, two improvement schemes are proposed: transforming the cylindrical high-voltage electrode of the microporous electrode into shapes with smaller radii of curvature, such as equilateral triangles and rectangular prisms, resulting in rectangular microporous electrodes and triangular pyramidal electrodes.

### 4.1. Rectangular Microporous Electrode

**Figure 7:**
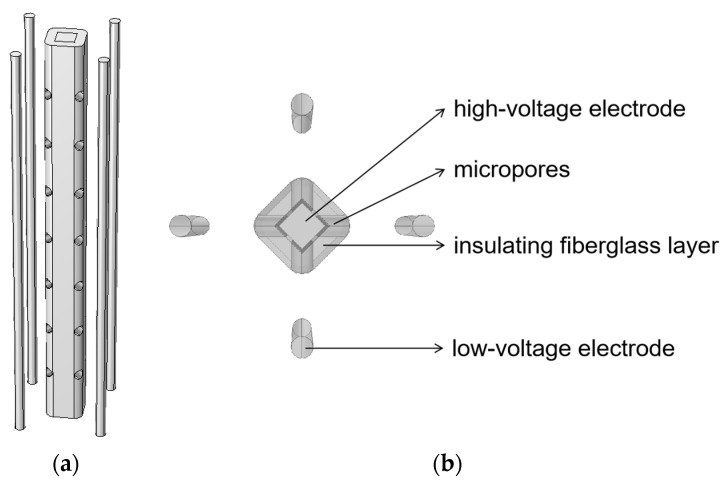
Rectangular microporous electrode model diagram: (**a**) overall view; (**b**) top view.

The structural model of the rectangular microporous electrode is shown in Figure 7. In this design, the high-voltage electrode of the microporous electrode is changed from a cylindrical shape to a rectangular prism, and stainless steel is used as the material. The high-voltage electrode is encased in an insulating layer of glass fiber, with four rows of micropores arranged along the four edges of the rectangular prism. Each row consists of seven evenly spaced cylindrical pores. The four edges of the high-voltage electrode are surrounded by four stainless steel rods which act as low-voltage electrodes. The dimensional parameters corresponding to the rectangular microporous electrode model are given in Table 3. The circuit and water parameters are the same as for the microporous electrode.

### 4.2. Triangular Pyramid Pore Electrode

**Figure 8:**
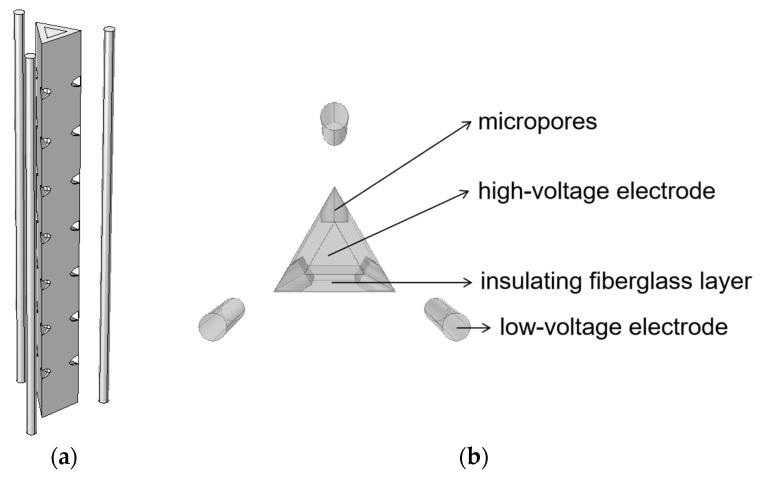
Triangular pyramid pore electrode model diagram: (**a**) overall view; (**b**) top view.

The structural model of the triangular pyramid pore electrode is shown in Figure 8. In this design, the high-voltage electrode of the microporous electrode is changed from a cylindrical shape to a triangular pyramidal shape, and stainless steel is used as the material. The high-voltage electrode is encased in an insulating layer of glass fiber, with three rows of micropores arranged along the three edges of the triangular pyramid. Each row consists of seven evenly spaced cylindrical pores. The three edges of the high-voltage electrode are surrounded by three stainless steel rods which act as low-voltage electrodes. The dimensional parameters for the triangular pyramidal microporous electrode model are given in Table 4. The circuit and water parameters are the same as for the microporous electrode.

## 5. Discussion

Having established the models for the rectangular microporous electrode and the triangular pyramidal microporous electrode, the same circuit and water parameters used in the microporous electrode calculation are applied to induce breakdown in both the rectangular and triangular pyramidal microporous electrodes. The electroacoustic parameters calculated for the rectangular microporous electrode, the triangular pyramidal microporous electrode, and the microporous electrode model from the previous section are summarized in Table 5. By performing simulation calculations for the microporous electrode, rectangular microporous electrode, and triangular pyramidal microporous electrode, the electroacoustic characteristic parameters for the three types of microporous electrode structures are obtained. The main electroacoustic parameters include the pre-breakdown time, the voltage at the moment of breakdown, the peak discharge current, and the shock wave sound pressure. Equations (4)–(6) can be used to derive the electroacoustic conversion efficiency from the total discharge energy in the liquid of the transmitting transducer to the shock wave energy.

From Table 5, it can be seen that, for the same circuit and water parameters, changing the high-voltage electrode of the microporous electrode from a cylindrical shape to a rectangular prism or a triangular pyramidal shape increases the curvature at the tip of the microporous high-voltage anode. This results in a stronger non-uniform electric field at the tip, making it easier to form a plasma channel. As a result, the pre-breakdown time of the rectangular microporous electrode and the triangular pyramidal microporous electrode is shorter than that of the cylindrical microporous electrode. The shortened pre-breakdown time leads to an increase in the breakdown voltage, which in turn generates a higher discharge current and a higher shock wave pressure. Furthermore, the electroacoustic conversion efficiency, which represents the conversion of the total discharge energy in the liquid of the transmitting transducer into shock wave energy, is also improved.

**Figure 9:**
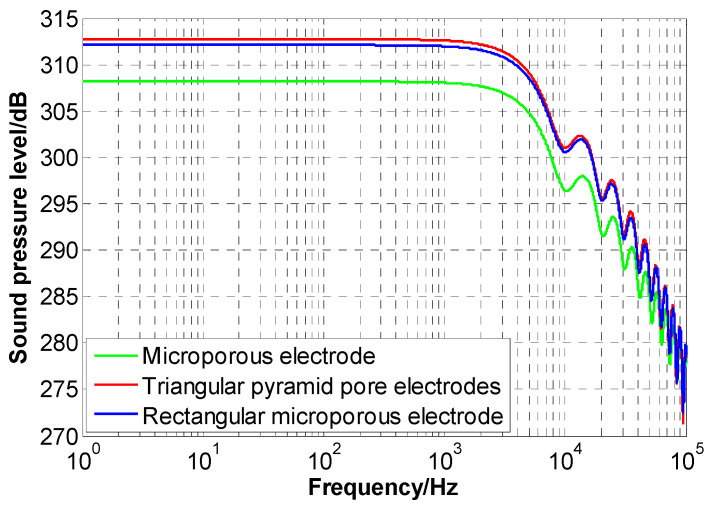
Sound pressure level vs. frequency graph for the micropore series electrodes.

Using Equation (2), the shock wave pressures of the microporous electrode, rectangular microporous electrode, and triangular pyramidal microporous electrode are converted into sound pressure levels, followed by an FFT (Fast Fourier Transformation) transformation to plot the sound pressure level versus frequency graph as shown in Figure 9. At constant frequencies, the shock wave sound pressure level of the triangular pyramidal microporous electrode is greater than that of the microporous electrode and the rectangular microporous electrode. In the 10 kHz range, the shock wave sound pressure level of the triangular pyramidal microporous electrode reaches 313 dB, and it exceeds 270 dB in the 100 kHz range. Therefore, optimizing the cylindrical high-voltage electrode of the microporous electrode to a tip-shaped electrode with a larger curvature significantly increases the amplitude of the shock wave.

Both the rectangular microporous electrode and the triangular pyramidal microporous electrode provide certain improvements over the original microporous electrode. However, the tip curvature of the triangular pyramid is greater than that of the rectangular prism, making the optimization effect of the triangular pyramid electrode more significant. The triangular pyramid microporous electrode has a shorter pre-breakdown time, higher breakdown voltage, stronger shock wave pressure, and higher discharge current. It also achieves higher electro-acoustic conversion efficiency.

Utilizing the triangular pyramidal microporous electrode as the electrode structure for a liquid–electric transducer can more effectively enhance its performance. It operates within a frequency band of 0 to 100 kHz and is capable of emitting high-energy shock waves. The excellent electroacoustic properties of the liquid–electric triangular pyramidal transducer further expand its application range in practical well-logging. At low frequencies with a strong shock wave pressure, the transducer can detect larger geological structures, which is advantageous for connecting extended-reach U-shaped wells and relief wells, facilitating early entry into target formations to improve recovery rates, and enhancing borehole smoothness. At high frequencies with strong shock wave pressure, it improves the resolution of geological structures, making it suitable for detecting microfractures, hydraulic fractures, small remaining oil pockets, and minor geological formations. Moreover, the compact size of the liquid–electric triangular pyramidal transducer fits within conventional drill collars, making it capable of operating in confined downhole spaces. It meets the surveying requirements beyond a hundred meters and enables precise descriptions of small geological bodies, which holds significant importance for applications in marine exploration, land exploration, clean energy, and emerging energy sectors.

## 6. Conclusions

Based on the traditional piezoelectric transducer used in logging, a liquid–electric micropore emission transducer was proposed. A model of the liquid–electric micropore emission transducer was constructed, and its electroacoustic characteristics were analyzed. Subsequently, two innovative optimization schemes for the micropore electrode structure were proposed: the triangular pyramid micropore electrode structure and the rectangular micropore electrode structure, and a comparison was made. The main conclusions are as follows:

(1) Traditional piezoelectric transducers are commonly used in logging, but due to the space constraints in the borehole, the frequency range and sound pressure intensity are limited, making them unsuitable for achieving the longer range and higher resolution required for next-generation sonic logging tools.
(2) A liquid–electric micropore emission transducer was proposed, and a breakdown discharge model for the micropore electrode in water was constructed. The electroacoustic characteristics were analyzed, revealing that the liquid–electric micropore emission transducer achieved a maximum shockwave pressure of 19.26 MPa.
(3) Both improved designs for the micropore electrode—rectangular micropore electrodes and triangular pyramid micropore electrodes—were found to enhance the performance of the micropore electrode. However, the triangular pyramid micropore electrode is better suited for logging applications, with a stronger shockwave pressure of 22.33 MPa and a broader frequency band, covering 0–100 kHz.
(4) The newly optimized liquid–electric triangular pyramid micropore electrode transducer can more effectively enhance the performance of emission transducers, significantly expanding the scope of logging applications. This has important implications for fields such as marine exploration, land-based exploration, clean energy, and new energy sources.

## Figures and Tables

**Table 1:** Circuit parameters and water parameters.

Circuit Parameters and Water	Parameters
Charging voltage	20 kV
Stored energy capacitor	15 μF
Equivalent inductance	8.18 μH
Equivalent resistance	0.22 Ω
Conductivity of tap water	0.07 S/m
Relative permittivity of tap water	81

**Table 2:** Microporous electrode dimension parameters.

Part of Microporous Electrode			Parameters
High-voltage electrode	Radius/mm		2.5
	Height/mm		80
	Micropores	Spacing/mm	10
		Pore diameter/mm	1
		Pore depth/mm	2.5
Low-voltage electrode	Radius/mm		1
	Height/mm		80
Insulating fiberglass layer	Radius/mm		5
	Height/mm		80

**Table 3:** Rectangular microporous electrode dimension parameters.

Part of Rectangular Microporous Electrode			Parameters
High-voltage electrode	Length/mm		3.535
	Height/mm		80
	Micropores	Spacing/mm	10
		Pore diameter/mm	1
		Pore depth/mm	2.5
Low-voltage electrode	Radius/mm		1
	Height/mm		80
Insulating fiberglass layer	Length/mm		7.07
	Height/mm		80

**Table 4:** Triangular pyramid pore electrode dimension parameters.

Part of Triangular Pyramid Pore Electrode			Parameters
High-voltage electrode	Length/mm		4.33
	Height/mm		80
	Micropores	Spacing/mm	10
		Pore diameter/mm	1
		Pore depth/mm	2.5
Low-voltage electrode	Radius/mm		1
	Height/mm		80
Insulating fiberglass layer	Length/mm		8.66
	Height/mm		80

**Table 5:** Electroacoustic characteristic parameters of the microporous electrode series structures.

Electrode Structure	Pre-Breakdown Time/μs	Breakdown Voltage/kV	Peak Discharge Current/kA	Maximum Shockwave Sound Pressure/MPa	Electroacoustic Conversion Efficiency/%
Micropore electrode	148.01	18.657	21.86	19.26	5.6539
Rectangular microporous electrode	46.77	19.549	22.76	21.22	6.1847
Triangular pyramid pore electrode	40.74	19.766	24.37	22.33	6.9646

## Data Availability

The original contributions presented in the study are included in the article; further inquiries can be directed to the corresponding author.
